# Basal and inducible Osterix expression reflect equine mesenchymal progenitor cell osteogenic capacity

**DOI:** 10.3389/fvets.2023.1125893

**Published:** 2023-03-23

**Authors:** Antonella Liza Pantaleoni Andrietti, Sushmitha S. Durgam, Brittany Naumann, Matthew Stewart

**Affiliations:** Department of Veterinary Clinical Medicine, University of Illinois, Urbana, IL, United States

**Keywords:** osteogenesis, mesenchymal stem cells, Runx2, Osterix, bone formation

## Abstract

**Introduction:**

Mesenchymal stem cells are characterized by their capacities for extensive proliferation through multiple passages and, classically, tri-lineage differentiation along osteogenic, chondrogenic and adipogenic lineages. This study was carried out to compare osteogenesis in equine bone marrow-, synovium- and adipose-derived cells, and to determine whether osteogenic capacity is reflected in the basal expression of the critical osteogenic transcription factors Runx2 and Osterix.

**Methods:**

Bone marrow, synovium and adipose tissue was collected from six healthy 2-year-old horses. Cells were isolated from these sources and expanded through two passages. Basal expression of Runx2 and Osterix was assessed in undifferentiated third passage cells, along with their response to osteogenic culture conditions.

**Results:**

Bone marrow-derived cells had significantly higher basal expression of Osterix, but not Runx2. In osteogenic medium, bone-marrow cells rapidly developed dense, multicellular aggregates that stained strongly for mineral and alkaline phosphatase activity. Synovial and adipose cell cultures showed far less matrix mineralization. Bone marrow cells significantly up-regulated alkaline phosphatase mRNA expression and enzymatic activity at 7 and 14 days. Alkaline phosphatase expression and activity were increased in adipose cultures after 14 days, although these values were less than in bone marrow cultures. There was no change in alkaline phosphatase in synovial cultures. In osteogenic medium, bone marrow cultures increased both Runx2 and Osterix mRNA expression significantly at 7 and 14 days. Expression of both transcription factors did not change in synovial or adipose cultures.

**Discussion:**

These results demonstrate that basal Osterix expression differs significantly in progenitor cells derived from different tissue sources and reflects the osteogenic potential of the cell populations.

## Introduction

Mesenchymal stem cells (MSC) are characterized by their capacities for extensive proliferation through multiple passages and multi-lineage differentiation; classically, along osteogenic, chondrogenic and adipogenic lineages (1, 2). Accepting these common features, the specific phenotypic and functional capacities of MSCs derived from different sources vary considerably, despite very similar isolation, *in vitro* expansion and differentiation protocols (3–9). The underlying mechanisms responsible for these lineage predispositions are not well understood. Therapeutic MSC applications derive from their ability to differentiate and contribute directly to tissue repair, regulate the activities of adjacent cells through trophic effects and/or immunomodulate host responses (10–16). During skeletal repair, osteo-progenitors from the periosteum and marrow cavity contribute directly to bone regeneration (17, 18). Strategies designed to stimulate endogenous osteoprogenitor activities or deliver exogenous stem cells to fracture sites have considerable potential to improve fracture repair by accelerating the time to skeletal stabilization. Self-evidently, therapeutic cells need to be capable of robust and rapid osteogenic differentiation for clinical efficacy.

Osteogenesis is one of the primary differentiation pathways used to characterize MSCs. The *in vitro* requirements for this process and informative phenotypic indices have been clearly defined, and transcriptional regulation of osteogenesis in developmental contexts has been well-characterized. Two transcription factors, Runx2 and Sp7/Osterix (OSX), are mandatory for this pathway, as clearly demonstrated in murine gene deletion models (19–21). Developmentally, Runx2 induces OSX expression and, collectively, these transcription factors drive expression of genes required for skeletogenesis (22).

This study was carried out to compare the osteogenic capabilities of three equine putative MSC populations, derived from bone marrow (BM), synovium (SYN) and adipose tissue (ADI), and to determine whether any osteogenic lineage predisposition is reflected in the expression of core osteogenic transcription factors under basal (non-induced) culture conditions. Both bone marrow- and adipose-derived MSCs are used in a wide range of clinical applications (23–26), while synovium-derived MSCs are representative of progenitors particularly predisposed to chondrogenic differentiation and have been applied experimentally for intra-articular therapy and articular cartilage repair (27, 28). The experiments were designed to test the hypothesis that basal and inducible Runx2 and OSX expression reflects the osteogenic capacity of equine progenitor populations.

## Materials and methods

### Bone marrow aspirate, synovium and adipose tissue collection

Bone marrow (BM), synovium (SYN) and adipose (ADI) tissue were collected from six healthy 2-year-old horses that were being euthanized at the termination of an unrelated study. The use of these horses for this study was approved by the Institutional Animal Care and Use Committee. Horses were sedated with 1.0 mg of xylazine/kg IV. Anesthesia was induced with 2.2 mg of ketamine/kg and 0.1 mg of diazepam/kg and maintained with 5% guaifenesin solution containing 1 mg of ketamine/L and 1 gm of xylazine/L.

To collect bone marrow, the skin over the tuber coxae was clipped and aseptically prepared. A stab incision was made through the skin with a #11 scalpel blade and 10–15 ml of bone marrow was aspirated through a Jamshidi biopsy needle into a syringe containing 1,000 IU of heparin. Following collection of bone marrow aspirates, all horses were euthanized with an intravenous injection of 104 mg of sodium pentobarbital/kg. Adipose tissue and synovium were collected immediately following euthanasia.

Adipose tissue was collected from the subcutaneous depot lateral to the tail head. The skin was clipped and disinfected, 8–10 g of adipose tissue was collected through a 10–15 cm skin incision and placed in a 50 ml polypropylene tube containing sterile phosphate buffered saline (PBS) solution. For synovium collection, the skin over the right radiocarpal joint was clipped, aseptically prepared and then reflected by sharp dissection to expose the dorsal aspect of the carpus. A transverse incision was made through the dorsoproximal aspect of the radiocarpal joint capsule and the synovial membrane was exposed by inverting the capsule. Approximately 2–3 g of synovium was excised from the inner surface of the capsule and placed into a 50 ml polypropylene tube containing sterile PBS solution.

### Cell isolation and monolayer expansion

Bone marrow aspirates were diluted with 10 ml of PBS and centrifuged at 300 g for 15 mins. The cell pellet was washed with PBS and re-centrifuged. The supernatant was removed, and the cell pellet was re-suspended with 0.8% ammonium chloride to lyse red blood cells. The remaining nucleated cells were pelleted by centrifugation, as above, re-suspended and cultured in Dulbecco's modified Eagle's medium (DMEM; Corning, Corning, NY), supplemented with 10% fetal bovine serum (FBS; GeminiBio, West Sacramento, CA) and 1% penicillin/streptomycin (BioWhittaker, Walkersville, MD; growth medium) until the primary monolayers reached 80% confluence.

Synovial tissue was digested in 0.25% trypsin/EDTA (Corning) at 37°C for 30 mins. Subsequently, the tissue was transferred to 0.1% collagenase (type II; Worthington Biochemical Corporation, Lakewood, NJ) in DMEM (10 ml of medium/gram of tissue) supplemented with 10% FBS and 2% penicillin/streptomycin for 2 h at 37°C in a shaking incubator. Adipose tissue was diced into small pieces and digested for 3 h at 37°C in 0.2 % collagenase (type II; Worthington) in DMEM (Gibco-ThermoFisher Scientific, Waltham, MA: 10 ml of medium/gram of tissue) and 2% penicillin/streptomycin (Gibco). After digestion, SYN and ADI cells were filtered through 40 μm mesh filters (Corning) and collected by centrifugation at 300 g for 10 mins. The numbers of primary ADI and SYN cells were determined with a hematocytometer and cellular viability was assessed by trypan blue exclusion. Primary SYN and ADI cells were seeded at 5 × 10^3^ cells/cm^2^ in 100 mm culture plates (Corning) and maintained in DMEM/10% FBS at 37°C in 5% CO_2_. The medium was changed three times per week.

When the primary BM, SYN and ADI cultures reached 80% confluence, the monolayers were lifted by brief 0.05% trypsin-EDTA (Gibco) digestion. The primary cell isolates were passaged twice, at initial seeding densities of 5 × 10^3^ cells/cm^2^, to enrich for highly and persistently proliferative progenitor cells and generate sufficient numbers of third passage cells for subsequent differentiation experiments.

### Osteogenic cultures

Cells were seeded at 2 × 10^4^ cells/cm^2^ in DMEM/10% FBS (control medium) and maintained until the monolayers were 70–80% confluent. Cultures for Alizarin Red, von Kossa and ALP staining, and for RNA isolation were seeded in six well plates, while cultures designated for ALP activity assays were seeded in 12 well plates (Corning). Control cultures remained in DMEM/10% FBS, while osteogenic cultures were transferred to control medium supplemented with 100 nM Dexamethasone (Sigma-Aldrich, St. Louis, MO), 10 mM β-Glycerophosphate (Sigma-Aldrich) and 50 μg/ml ascorbic acid (Wako Pure Chemical Industries, Japan). The responses of BM, SYN and ADI cells to osteogenic medium were monitored daily *via* light microscopy, representative images were recorded (Leica Microsystems, Leica Application Suite—LAS—version 2.6.R1) and phenotypic transition was assessed after 7 and 14 days, as detailed below.

### Alizarin Red staining

After 7 and 14 days, the monolayers were rinsed with PBS, fixed in 10% formalin for 30 mins, then washed three times with distilled water. One ml of fresh 2% Alizarin Red (Sigma-Aldrich) solution (pH 4.1) was added to each well. Following incubation at room temperature for 20 mins on a shaking platform, the stain was removed, and the cells were washed with distilled water until the rinse solution was clear. Mineral deposits within the cell layers were stained bright red. Representative pictures of stained monolayers were obtained, as above.

### Von Kossa staining

Von Kossa stain (American MasterTech, Lodi, CA) was used to identify ionized phosphate in basal and osteogenic cultures. Following 30 mins fixation with 10% formalin, the cell layers were washed 2–3 times with distilled water. One ml of 5% silver nitrate solution was added to each well and exposed to a strong light for 30 mins. The cell layers were washed 2–3 times with distilled water and 1 ml of 5% sodium thiosulfate was added for 5 mins to remove excess silver salts. The cell layers were washed 2–3 times with distilled water. Finally, a neutral red solution was added for 5 mins as a counterstain. Calcium deposits in the extracellular matrices were evident as dark brown or black deposits within the cell aggregates. Representative images of stained monolayers were obtained, as above.

### Alkaline phosphatase staining

After 7 and 14 days, cell layers were fixed with citrate-acetone-formaldehyde fixative solution for 1 min followed by three washes with distilled water. An alkaline dye consisting of a diazonium salt solution and naphthol AS-BI alkaline solution (Procedure No. 86, AP, leukocyte; Sigma Aldrich) was added to the cell layer and incubated in reduced light conditions at room temperature for 15 min. The monolayers were washed again with distilled water, and the cell layers were then counterstained with neutral red solution for 5 min. Cells exhibiting ALP activity were marked by blue staining. Representative pictures of the stained monolayers were obtained by microscopy and digital photography.

### Alkaline phosphatase enzymatic activity

ALP activity was assessed in triplicate samples of control and osteogenic cultures from each donor. At days 7 and 14, the cells were harvested in 1 ml of lysis buffer containing 20 mM Tris HCl, 150 mM NaCl and 1% Triton X-100 (Sigma-Aldrich). Each sample was homogenized using an IKA Labortechnik T 25 basic homogenizer (Janke and Kunkel GmbH and Co, Staufen, Germany), centrifuged at 2,500 rpm for 15 mins at 4°C and kept on ice for 30 mins. Two 100 μl aliquots of the lysates were collected for DNA measurements (see below). The supernatants were assayed for ALP activity using an AP assay kit (Wako), following the manufacturer's instructions. The concentration of *p*-Nitrophenol was measured at 405 nm wavelength (FLUOstar OPTIMA, BMG, Lab Technologies, Cary, NC). The relative activity in each lysate was normalized to DNA content (see below).

### DNA measurement

DNA content was used as a surrogate indicator of cell number to normalize ALP activity data. DNA was measured using the Pico Green DNA kit (Quanti-iT^TM^ PicoGreen dsDNA, Invitrogen). Serial dilutions of calf thymus DNA were used to generate a standard curve. Duplicate 100 μl aliquots of each lysate were diluted 1:5 in TE buffer (10 mM Tris-HCl, 1 mM EDTA, pH 7.5) and transferred to black 96-well microplates (Corning). One microliter of Pico Green reagent diluted in 200 μl of TE buffer was added to each sample. Following 5 mins incubation in a lightproof container, fluorescence was measured at 485 nm wavelength (FLUOstar OPTIMA).

### RNA isolation and quantitative PCR analyses

Total RNA was isolated using TRIzol^®^ (Invitrogen Corporation), according to the manufacturer's recommended protocol. The lysates were homogenized prior to chloroform addition. One microgram of total RNA was reverse-transcribed, using oligo dT to prime the reactions (Superscript^TM^ First-Strand Synthesis System for RT-PCR, Invitrogen Life technologies, Carlsbad, CA).

ALP, Runx2 and OSX transcript levels was measured by quantitative real-time PCR (qPCR), normalized to expression of the reference gene, elongation factor-1 alpha (EF1-α). The primers used for qPCR analysis are listed in Table 1. For all primers, the optimum annealing temperature was determined to be 57.7°C. During initial primer optimization trials, the amplicons were sequenced to ensure that the correct transcript was being amplified. Quantitative PCR was performed using 5 μl of diluted cDNA template (1:10 dilution) combined with 20 μl of a mixture composed of 12.5 μl 1 × SYBR Green Supermix (Bio-Rad Laboratories, Hercules, CA), 1 μl each of the 10 μM forward and reverse primer stocks and 5.5 μl DNase/RNase-free water in a 96-well microplate. Each sample was run in triplicate. The PCR reactions were run in a BioRad iCycler iQ (BioRad) using the following conditions: initial denaturation for 3 mins at 95°C, 40 cycles of denaturation at 95°C for 10 s, annealing temperature of 57.7°C for 30 s and polymerase extension at 72°C for 20 s. The presence of a single amplicon was monitored by melting curve analyses. The relative expression for each target gene was calculated using the comparative ΔCt method (29). For analyses of basal Runx2 and OSX expression in third passage cells, the mean level of BM expression in six donor samples was assigned a value of “1.” Within each data set from the control vs. osteogenic cultures, the “BM Day 7 Control” threshold cycle value in each horse was designated as “1” and the data from other times, cell sources and culture medium groups were adjusted accordingly.

**Table 1:** PCR primer sequences.

**Gene (size)**	**Sense primer**	**Annealing temperature**
	**Antisense primer**	
Runx2 (177 bp)	5′CAGACCAGCAGCACTCCATA (1,315)	57.7°C
	5′CAGCGTCAACACCATCATTC (1,492)	
Osterix (207 bp)	5′GGCTATGCCAATGACTACCC	57.7°C
	5′GGTGAGATGCCTGCATGGA	
ALP (221 bp)	5′TGGGGTGAAGGCTAATGAGG (357)	57.7°C
	5′GGCATCTCGTTGTCCGAGTA (578)	
EF1-α (328 bp)	5′CCCGGACACAGAGACTTCAT (48)	57.7°C
	5′AGCATGTTGTCACCATTCCA (376)	

### Statistical analyses

Mean ± SE values were calculated for each quantitative outcome measure. Differences in the basal expression of Runx2, OSX and ALP transcripts and ALP activities in undifferentiated third passage BM, SYN and ADI cells were assessed by one-way repeated measures ANOVA. The response of each cell type to osteogenic medium (at days 7 and 14), the comparative expression of ALP, Runx 2 and OSX mRNAs under basal (control) and osteogenic conditions were assessed by two-way repeated measures ANOVA. As required, Bonferroni's *post hoc* tests were applied to identify specific significant pair-wise differences. In all analyses, *P* values < 0.05 were considered significant. Statistical analyses were carried out using GraphPad Prism version 5.00 (GraphPad Software, San Diego California USA).

## Results

### Basal expression of Runx2 and OSX transcripts

**Figure 1:**
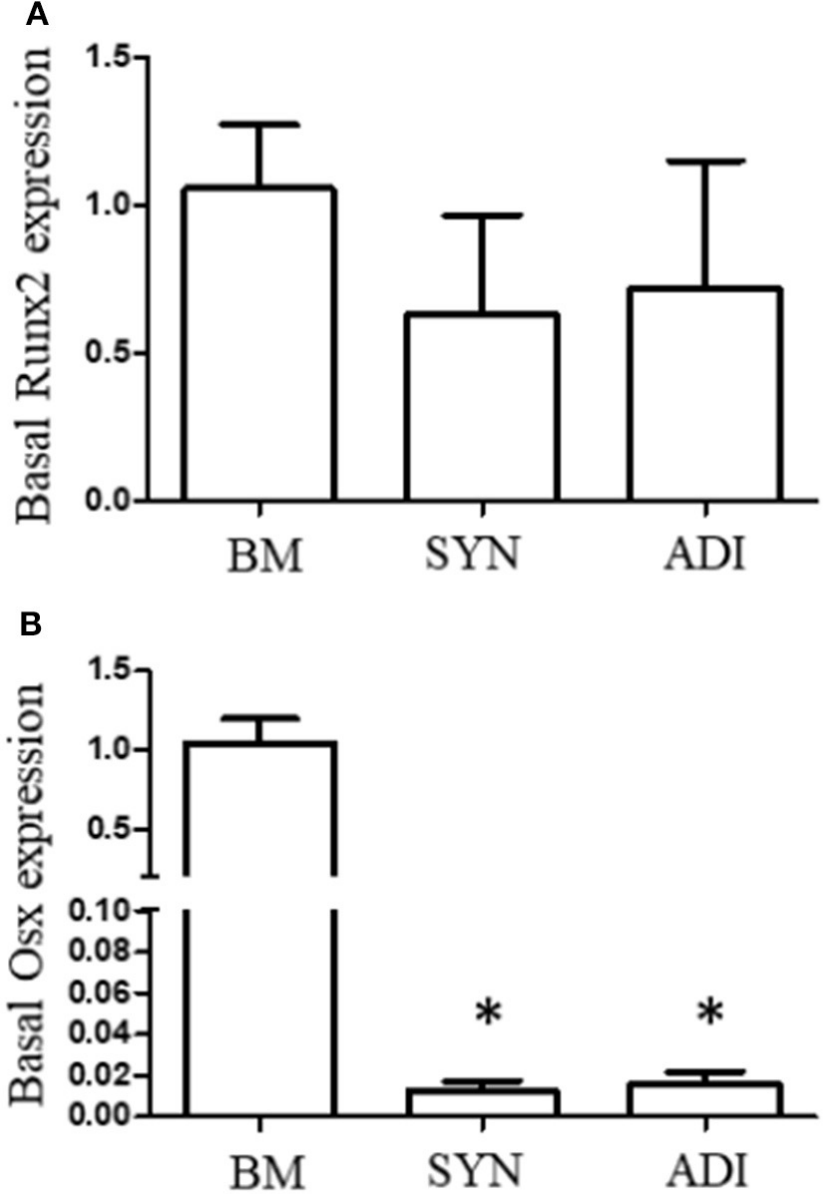
Basal expression of Runx2 **(A)** and Osterix **(B)** mRNAs in bone marrow- (BM), synovial- (SYN) and adipose- (ADI) third passage cells in control medium. Mean expression levels in BM samples were set at “1” in each analysis. Asterisks indicate mean + SE values significantly different from BM levels of expression (ANOVA *n* = 6; *P* < 0.05).

Under basal conditions, Runx2 expression was not different between the three cell types. In contrast, basal OSX mRNA expression was significantly higher, ~100-fold, in BM cells than in SYN and ADI cells (Figure 1).

### Monolayer culture responses to osteogenic medium

**Figure 2:**
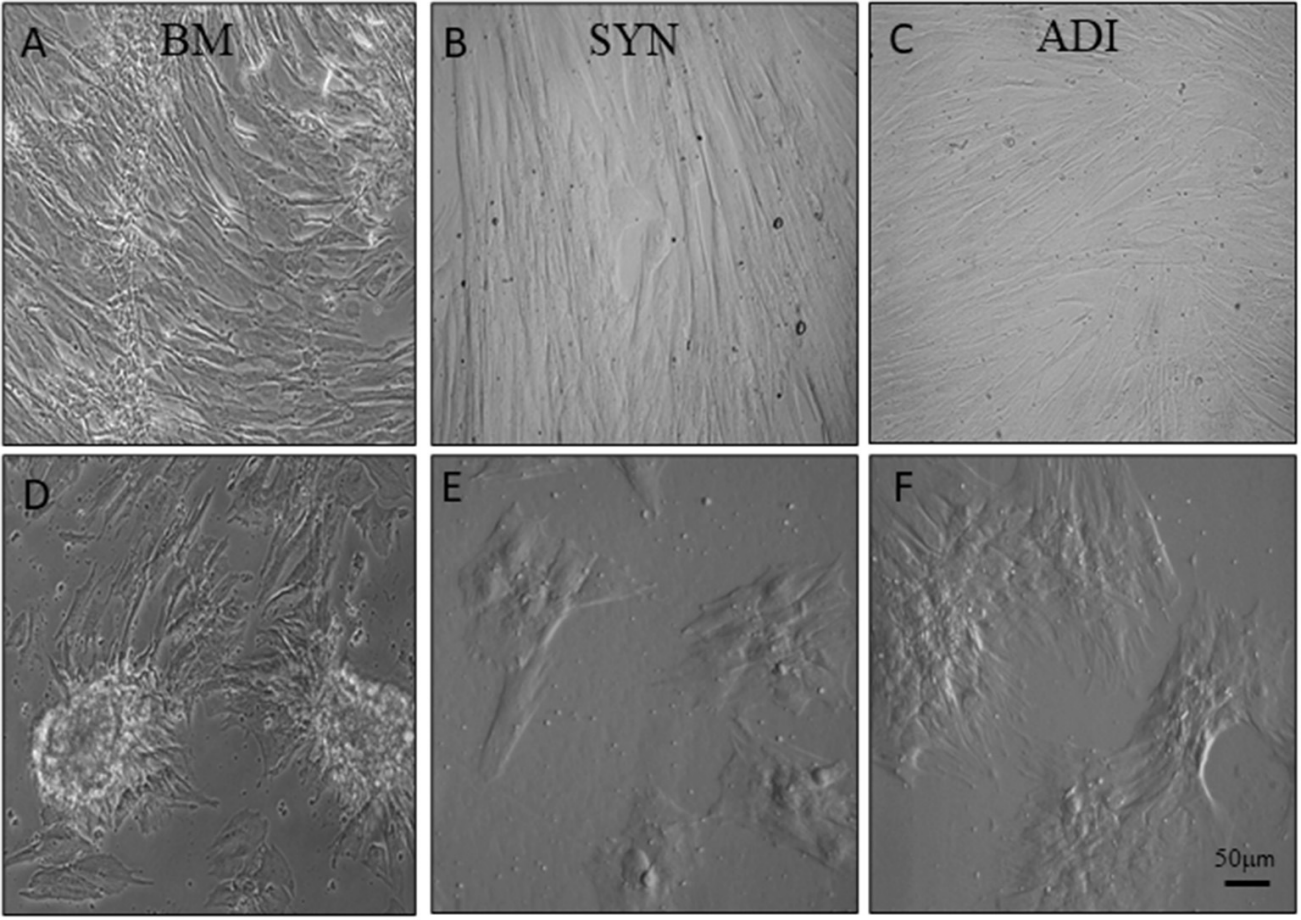
Representative microscopic images of bone marrow- [BM: **(A, D)**], synovial- [SYN: **(B, E)**] and adipose- [ADI: **(C, F)**] third passage cells in control **(A–C)** and osteogenic **(D–F)** medium after seven days.

Cultures in control medium maintained a flattened morphology throughout the 14-day time frame of the experiments (Figures 2A–C). When BM cells were transferred to osteogenic medium, the cells rapidly aggregated to form highly refractile, multicellular clusters that were clearly evident within 7 days. In contrast, cell aggregation in SYN and ADI cultures was noticeably slower in onset and did not form multilayered aggregates characteristic of BM cultures (Figures 2D–F).

### Matrix mineralization

**Figure 3:**
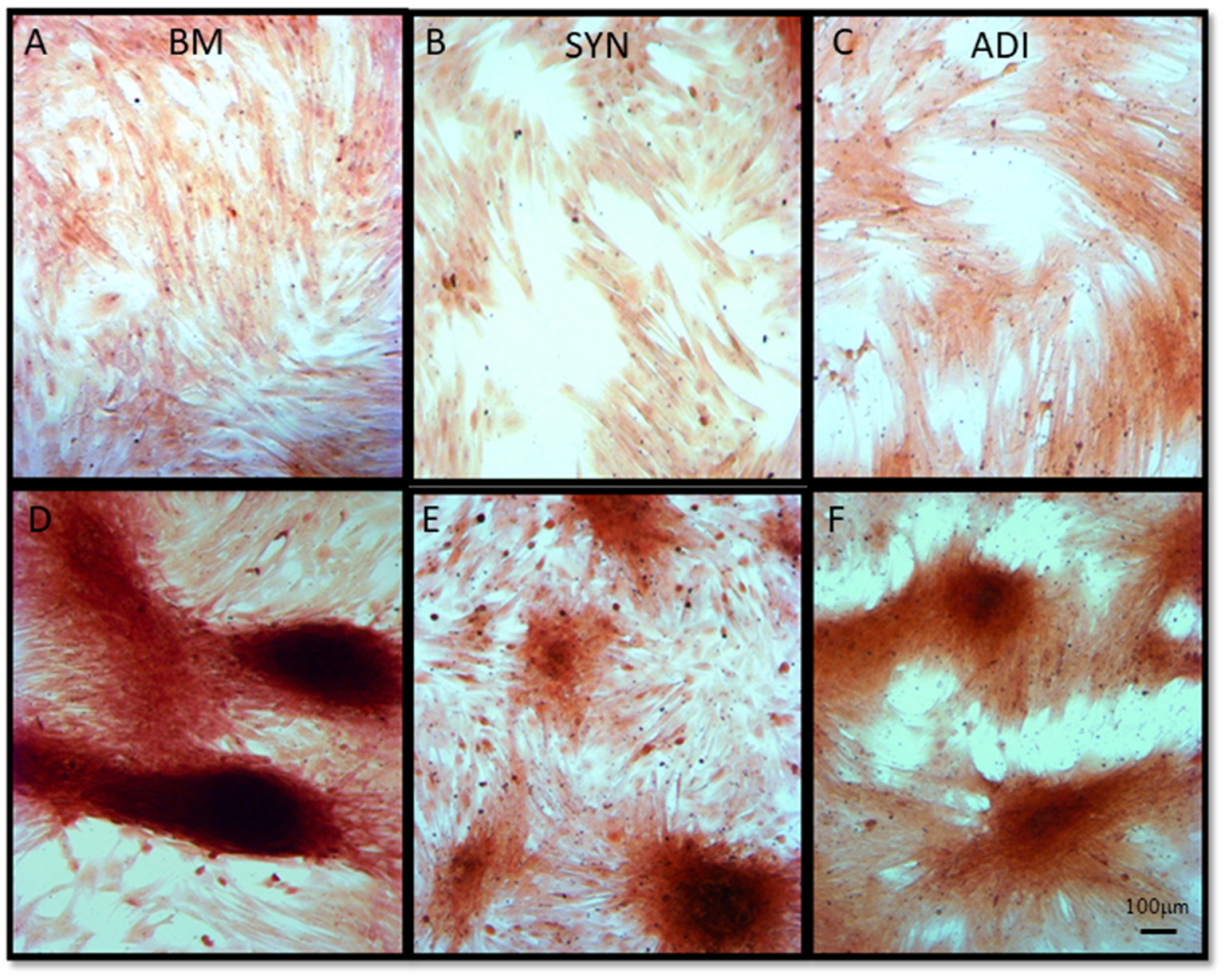
Representative microscopic images of bone marrow- [BM: **(A, D)**], synovial- [SYN: **(B, E)**] and adipose- [ADI: **(C, F)**] third passage cells in control **(A–C)** and osteogenic **(D–F)** medium after 14 days, stained with Alizarin Red solution to demonstrate the presence of ionized calcium deposition in basal **(A–C)** and osteogenic **(D–F)** cultures.

**Figure 4:**
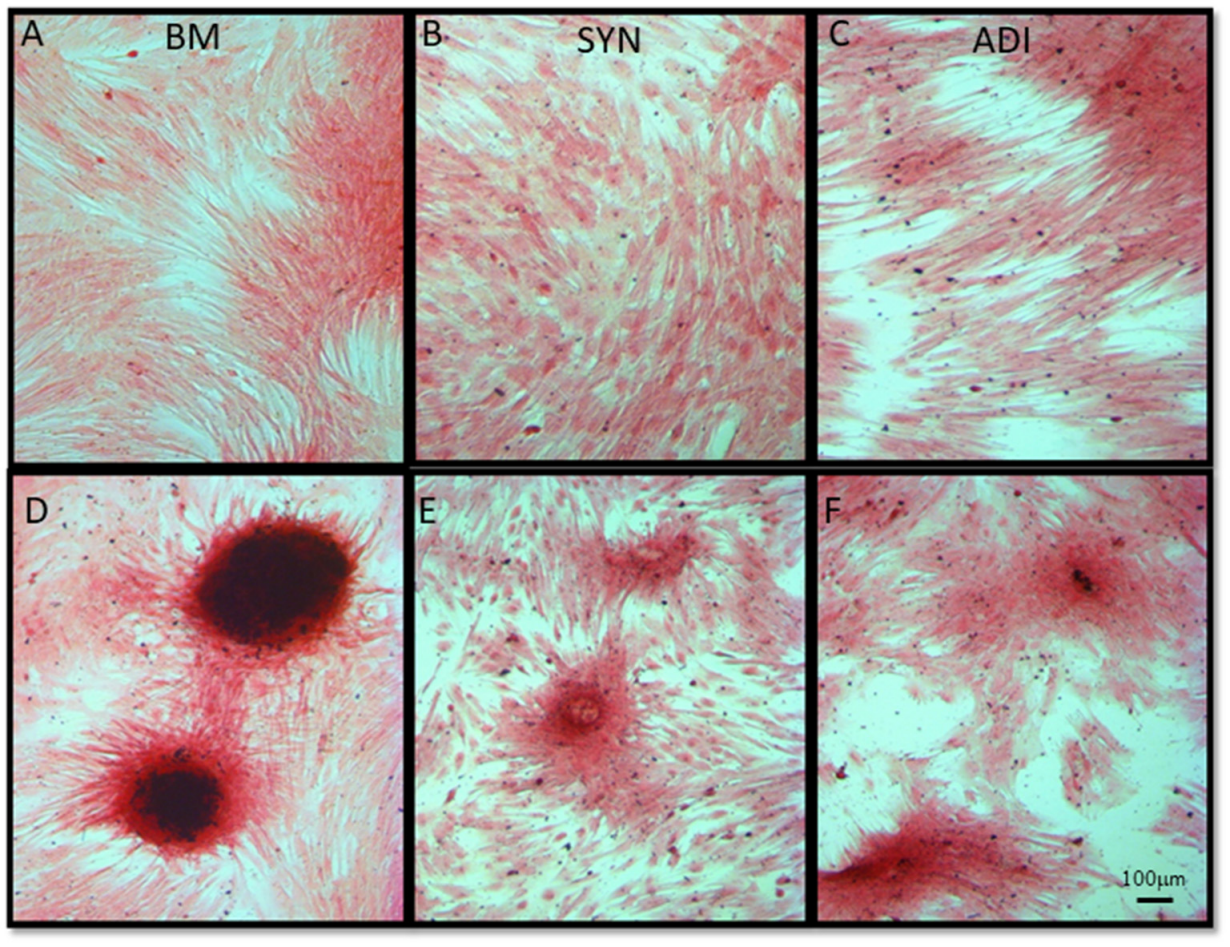
Representative microscopic images of bone marrow- [BM: **(A, D)**], synovial- [SYN: **(B, E)**] and adipose- [ADI: **(C, F)**] third passage cells in control **(A–C)** and osteogenic **(D–F)** medium after 14 days, stained with von Kossa stain to demonstrate the presence of ionized phosphate deposition in basal **(A–C)** and osteogenic **(D–F)** cultures.

Alizarin Red staining of the control cultures was minimal at both time points (Figures 3A–C). The cellular aggregates in the osteogenic BM cultures showed intense stain uptake at both day 7 and 14 (Figure 3D). Alizarin Red staining of osteogenic SYN and ADI cultures was restricted to the cell aggregates and was less intense than seen in BM cultures (Figures 3E, F). This was also evident in von Kossa-stained cultures. There was little or no stain uptake in control cultures (Figures 4A–C). In osteogenic BM cultures, the multicellular aggregates stained strongly for ionized phosphate by day 14 (Figure 4D), whereas phosphate deposition in osteogenic ADI and SYN cultures was minimal (Figures 4E, F).

### Alkaline phosphatase induction and activity

**Figure 5:**
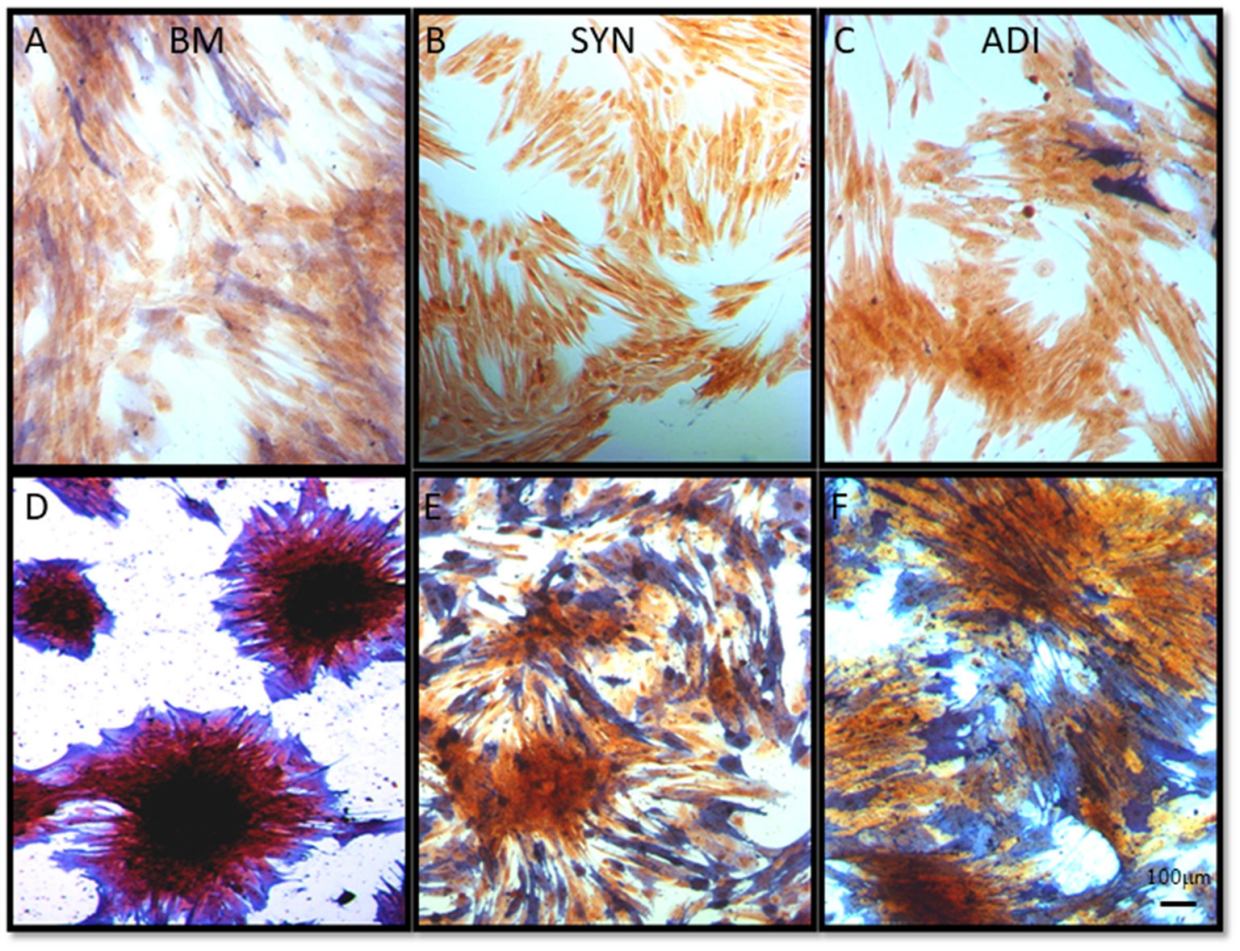
Representative microscopic images of bone marrow- [BM: **(A, D)**], synovial- [SYN: **(B, E)**] and adipose- [ADI: **(C, F)**] third passage cells in control **(A–C)** and osteogenic **(D–F)** medium after 14 days, stained with ALP solution to demonstrate the presence of cell-associated ALP activity.

Staining of control cultures for ALP activity demonstrated very faint and diffuse ALP signal in both the BM and ADI cultures. No stain was detectable in the SYN cultures (Figures 5A–C). Under osteogenic conditions, intense ALP activity was present within and immediately around the cell aggregates in BM cultures, whereas staining in the SYN and ADI monolayers was less intense and more diffusely distributed (Figures 5D–F).

**Figure 6:**
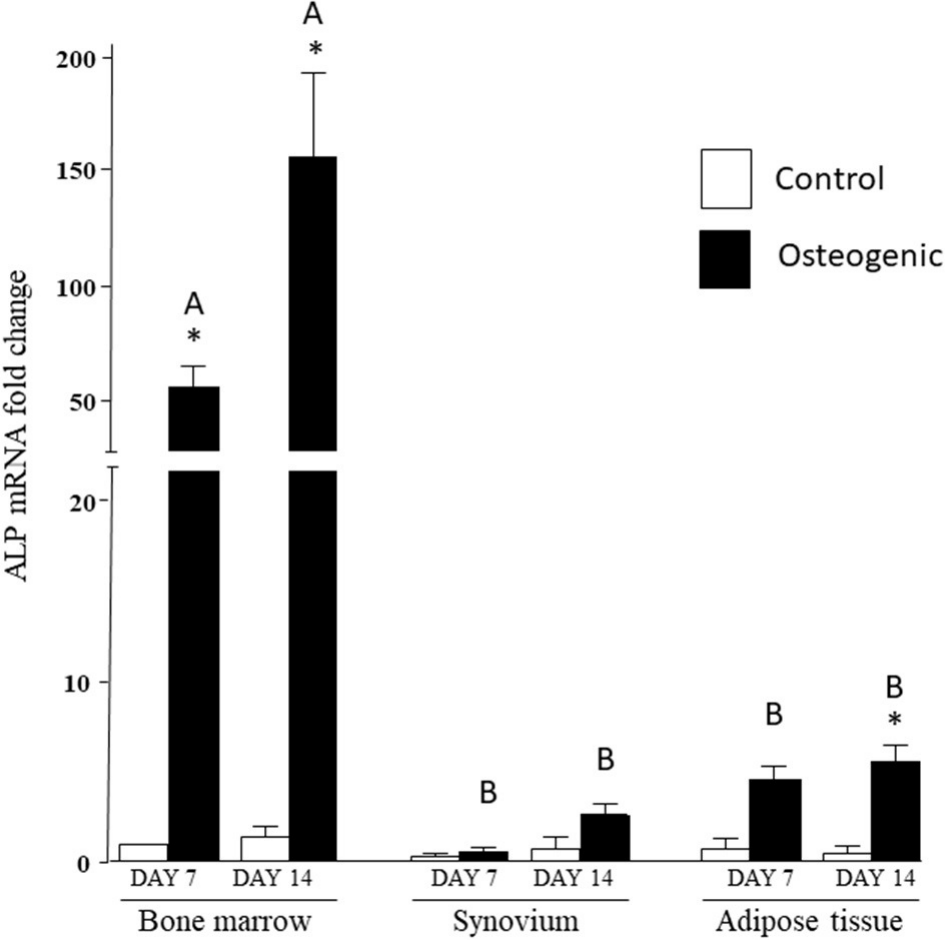
ALP mRNA expression in bone marrow-, synovium- and adipose tissue-derived cells in control (white columns) or osteogenic (black columns) medium after 7 or 14 days. Within each cell type, asterisks indicate mean + SE values significantly different from control levels of expression. Columns designated with different upper-case letters are significantly different in osteogenic cultures (two-way ANOVA *n* = 6; *P* < 0.05).

In control medium, ALP mRNA levels were statistically similar in the three cell groups at both time points. In osteogenic SYN cultures, ALP induction did not reach statistical significance, while ALP induction in ADI cultures was significant at day 14. ALP induction in BM osteogenic cultures was significant at both time points and significantly greater (100+ fold increase) than SYN and ADI culture values (Figure 6).

**Figure 7:**
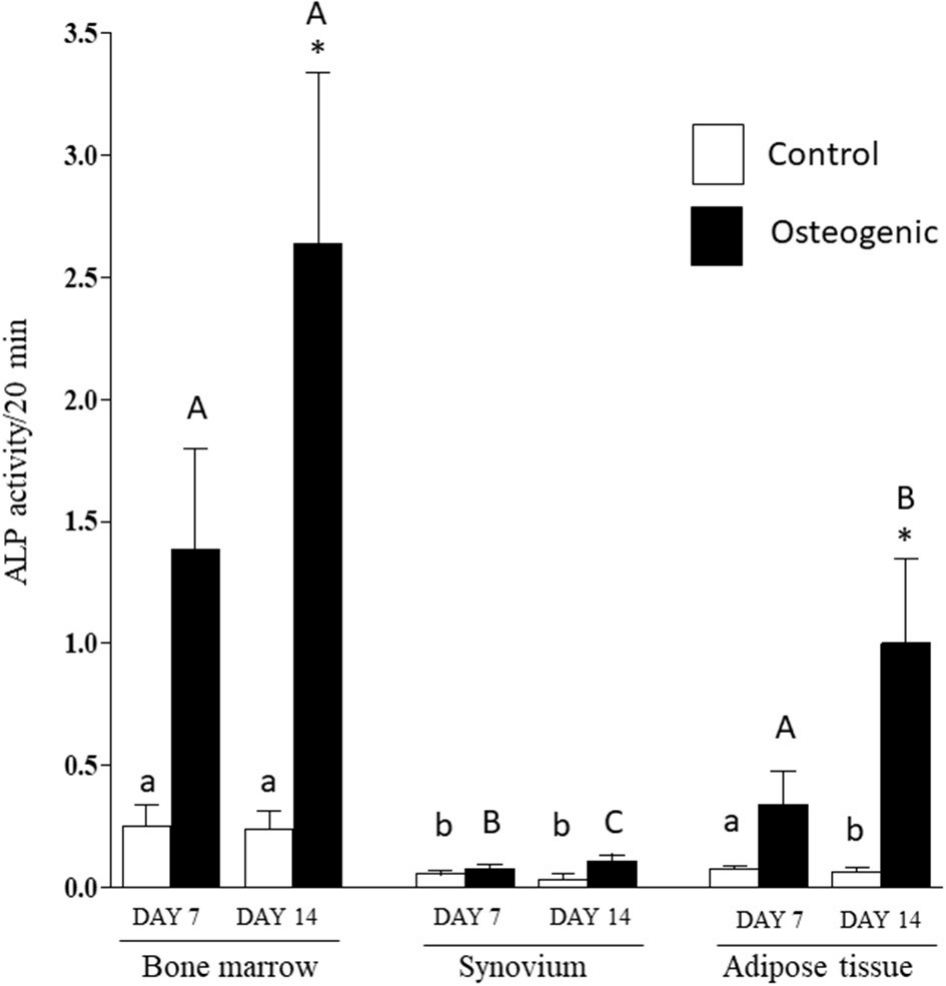
ALP enzymatic activities in bone marrow-, synovium- and adipose tissue-derived cells in control (white columns) or osteogenic (black columns) medium after 7 or 14 days. Within each cell type, asterisks indicate mean + SE values significantly different from control levels of expression Columns designated with different lower-case letters are significantly different in control cultures. Columns designated with different upper-case letters are significantly different in osteogenic cultures (two-way ANOVA *n* = 6; *P* < 0.05).

Comparative ALP enzymatic activities are presented in Figure 7. The differential activities were less substantive than the transcriptional analyses but followed similar profiles. In control medium, basal ALP activity was significantly higher in BM cultures than in SYN (both time points) and ADI (day 14) cultures. In osteogenic medium, ALP activity increased significantly (~10-fold) by day 14 in BM cultures. ALP activity was also significantly increased in osteogenic ADI cultures, although activity in these cells was still significantly lower than in the corresponding BM cultures. There was no increase in ALP activity in SYN cultures maintained in osteogenic medium.

### Comparative expression of Runx2 and OSX transcripts

**Figure 8:**
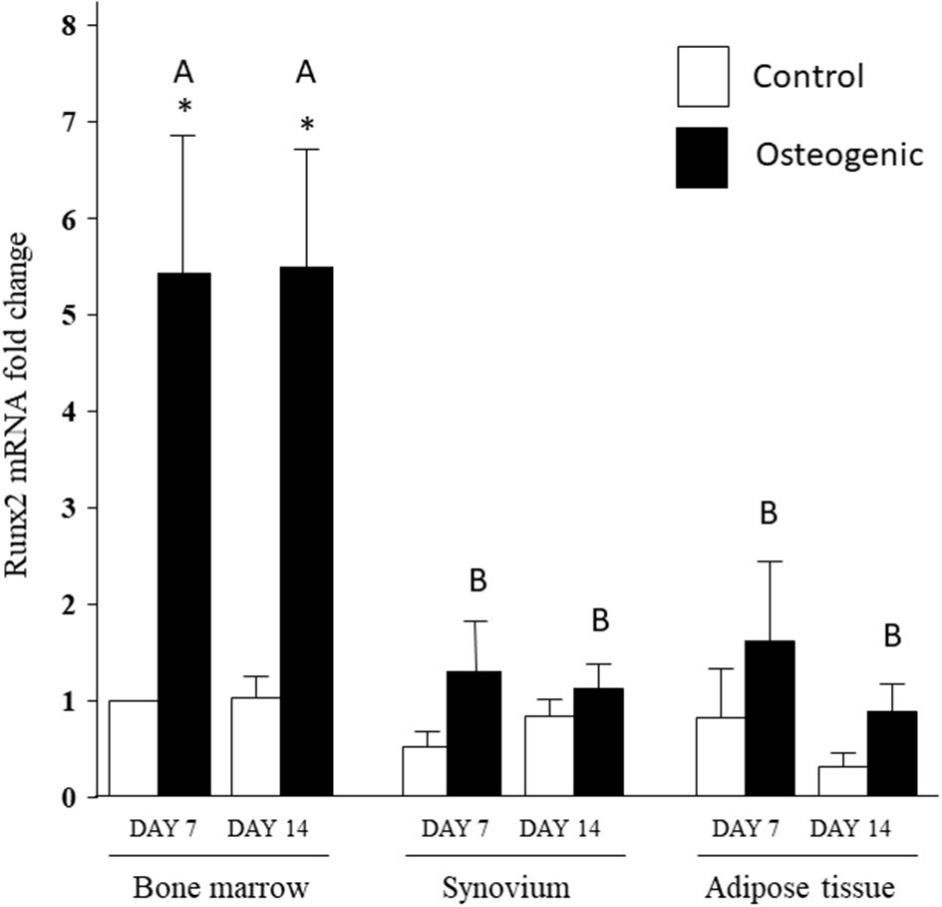
Runx2 mRNA expression in bone marrow-, synovium- and adipose tissue- derived cells in control (white columns) or osteogenic (black columns) medium after 7 or 14 days. Within each cell type, asterisks indicate mean + SE values significantly different from control levels of expression. Columns designated with different upper-case letters are significantly different in osteogenic cultures (two-way ANOVA *n* = 6; *P* < 0.05).

In control medium, Runx2 mRNA expression was stable over the 14 days of culture and remained similar across the three cell types (Figure 8). In osteogenic medium, Runx2 mRNA expression increased significantly in BM cells (~5-fold) at both time points. The slight increases in Runx2 transcript levels detected in SYN and ADI cultures were not statistically significant. Runx2 up-regulation in osteogenic BM cultures was significantly higher than Runx2 expression in osteogenic SYN or ADI cultures at both time points (Figure 8).

**Figure 9:**
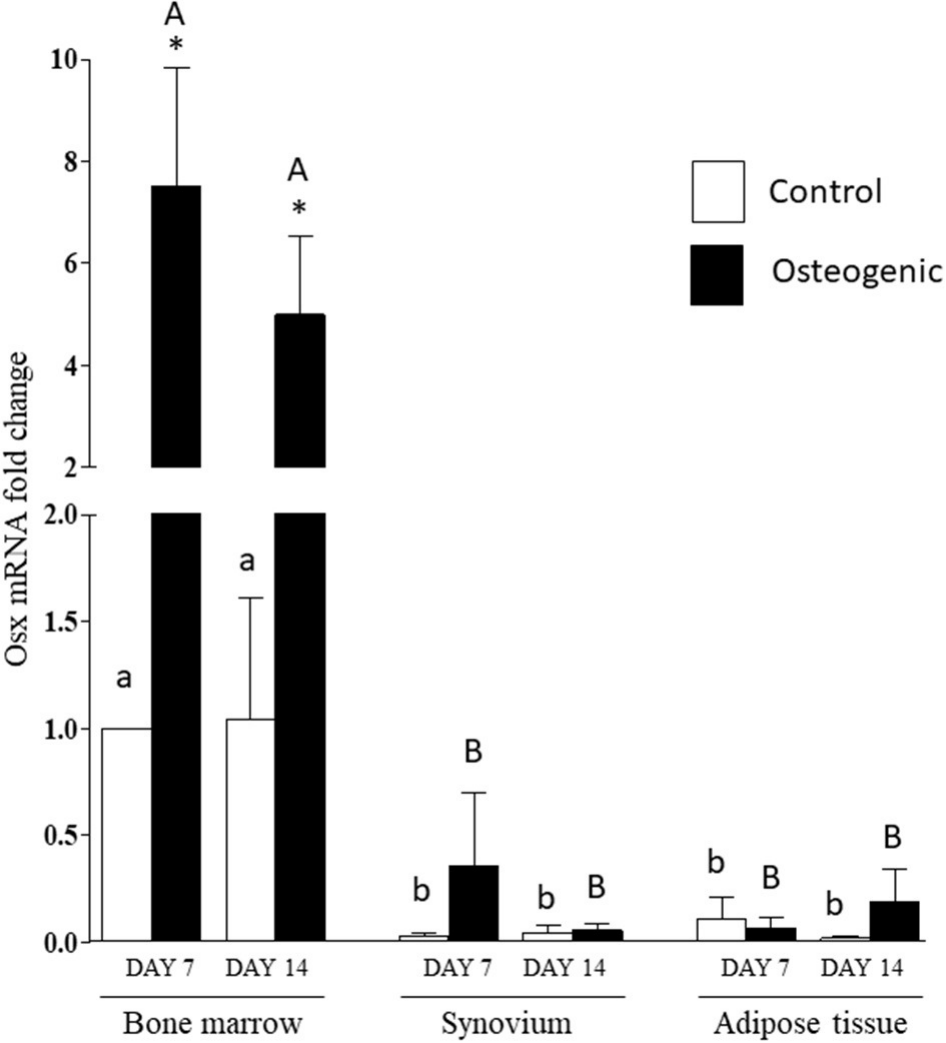
OSX mRNA expression in bone marrow-, synovium- and adipose tissue- derived cells in control (white columns) or osteogenic (black columns) medium after 7 or 14 days. Within each cell type, asterisks indicate mean + SE values significantly different from control levels of expression columns designated with different lower-case letters are significantly different in control cultures. Columns designated with different upper-case letters are significantly different in osteogenic cultures (two-way ANOVA *n* = 6; *P* < 0.05).

The significant differences in basal OSX mRNA expression were maintained in control cultures throughout the 14 days of the experiments (Figure 9). Osterix expression increased significantly in osteogenic BM cultures. In contrast, there were negligible changes in OSX expression in SYN and ADI cell osteogenic cultures (Figure 9). By day 14, mean OSX mRNA levels in osteogenic cultures were ~100-fold higher in BM cells than in SYN or ADI cells.

## Discussion

This study was carried out to assess the comparative osteogenic capabilities of progenitor populations derived from equine bone marrow, synovium and adipose tissue, and to determine whether any “pre-differentiation” lineage predisposition exists, as reflected in the expression of core osteogenic transcription factors. The study addressed the hypothesis that basal Runx2 and OSX expression reflects the osteogenic capacity of MSC populations. Not surprisingly, given the proximity of bone marrow-derived progenitors to sites of bone homeostasis, BM-MSCs were far more capable of osteogenic differentiation than cells isolated from synovium or adipose tissue within the 14-day time frame of the differentiation experiments, as indicated by significant differences matrix mineralization, ALP induction and activity. These observations do not preclude the possibility that osteogenic ADI and SYN cultures might have expressed osteogenic phenotypes more robustly, with longer times in culture.

Surprisingly, Runx2 expression under basal conditions did not differ between the cell groups at any time point. In this respect, our hypothesis was disproved. In marked contrast, basal OSX expression was substantially higher in BM cells and this differential expression profile was maintained in control cultures during the subsequent osteogenesis phase of the analyses. Further, the significant up-regulation of OSX mRNA levels in BM cells exposed to osteogenic medium did not occur in the other cell groups. These differential OSX expression profiles do support the hypothesis and suggest that screening for basal OSX expression could be used to identify cell populations with high osteogenic potentials. It also supports the use of OSX induction or expression strategies to increase or induce the osteogenic differentiation in cell populations for cell-based therapeutics, as has been experimentally demonstrated in bone marrow stromal cells (30).

Our experimental protocol did not include surface marker-based cell sorting or immunophenotyping to isolate or enrich for MSCs. We did apply prolonged, multi-passage *in vitro* expansion, using initially low seeding densities, to enrich our experimental populations for cells capable of sustained proliferation over many population doublings. Ranera et al. used isolation and expansion protocols almost identical to those used in the current study to demonstrate very similar immunophenotypes in third passage equine adipose- and bone marrow-derived cell populations, characteristic of MSCs (31). Heo et al. performed similar analyses in human progenitor cell populations, also using very similar isolation and expansion protocols, with the same finding (32). Unquestionably, our initial isolates from all three sources contained heterologous cell populations, however, the outcomes of the above-mentioned studies indicate that progenitor enrichment is achieved through multi-passage proliferative expansion of primary isolates.

Developmentally, OSX transcriptional expression is directly controlled by Runx2 trans-activity (19, 21, 22). Our results suggest that this regulatory pathway is not dominant in postnatal stem cell populations, and that factors distinct from Runx2 regulate OSX expression in adult progenitor populations. Both BMP and TGF-β signaling pathways upregulate OSX *via* a Runx2-independent transcriptional pathway, and Wnt and FGF signaling also impact OSX expression (33–36). It is plausible that differences in intrinsic signaling activities through one or more of these pathways determine the intrinsic osteogenic potentials of stem cell populations derived from different tissues, reflected in the levels of basal and inducible OSX expression (37).

The results of this study highlight the somewhat qualitative aspect of MSC phenotypic assessment through monolayer culture staining, as is commonly performed to demonstrate tri-lineage potential of putative MSC isolates. We assessed the ability of each cell type to undergo osteogenesis, using commonly used matrix staining protocols and statistically significant increases in Runx2, OSX and ALP expression or activity as criteria for differentiation. As expected, BM-MSCs were capable of robust and consistent osteogenic differentiation by all criteria used within the 14-day period of the experiments. In fact, these responses were clearly evident in BM cultures by day 7. ADI cell cultures maintained under osteogenic conditions for 14 days also exhibited some focal calcium deposition and ALP localization in the monolayer assays and significantly up-regulated ALP activity, but these responses were markedly less than were seen in BM cultures. More telling, von Kossa staining was negligible in ADI osteogenic cultures, and Runx2 and OSX expression did not increase significantly. SYN cell cultures also showed some matrix mineralization at day 14, along with a diffuse increase in ALP localization; however, by von Kossa staining and all quantitative assays, the SYN cells did not undergo significant osteogenesis within the 14-day time frame of the experiments. These outcomes are consistent with previous comparisons of the relative osteogenic capacities of putative stem cell populations (3–9). The collective outcomes of these analyses emphasize the need for multi-assay panels for rigorous assessments of MSC lineage commitment. Although culture staining and ALP assays are straightforward and inexpensive protocols, monolayer stain uptake and ALP induction can occur in the absence of other, more lineage-specific osteogenic transitions.

Regardless of the specific mechanism(s) that distinguishes the osteogenic potentials of bone marrow, synovial and adipose progenitor populations, these results emphasize that stem cell populations from specific tissue and fluid sources retain source-specific lineage potentials and predispositions. This observation needs to be taken into account with any anticipated clinical utilization of MSCs.

## Data availability statement

The original contributions presented in the study are included in the article/supplementary material, further inquiries can be directed to the corresponding author.

## Ethics statement

The animal study was reviewed and approved by IACUC, University of Illinois at Urbana-Champaign.

## Author contributions

AA, SD, BN, and MS contributed to tissue collection, cell isolation, and experimental protocol optimization. AA and BN were primarily responsible for cell culture maintenance, sample collection, outcome assays, and data collection. SD and MS were primarily responsible for data analyses. AA and MS were primarily responsible for manuscript development. All authors contributed to the article and approved the submitted version.
